# IgG4-Related Disease, the Malignancy Mimicker: Case Series from Bahrain

**DOI:** 10.1155/2018/4057024

**Published:** 2018-10-28

**Authors:** Majeed Haider, Fatima Haji, Osama Alalwan, Eman Aljufairi, Tejal S. Shah

**Affiliations:** ^1^Chief Resident, Rheumatology Unit, Salmaniya Medical Hospital, Manama, Bahrain; ^2^Consultant Rheumatologist, Rheumatology Unit, Salmaniya Medical Hospital, Manama, Bahrain; ^3^Consultant Pathologist, Pathology Unit, Salmaniya Medical Hospital, Manama, Bahrain; ^4^Consultant Diagnostic Radiologist, Radiology Unit, Salmaniya Medical Hospital, Manama, Bahrain

## Abstract

IgG4-related disease is an evolving immune-mediated condition. The hallmark of this condition is IgG4(+) plasma cells infiltration of the affected organs accompanied by a variable degree of fibrosis and occasionally elevated serum IgG4 level. It links many conditions that were once recognized as isolated unrelated idiopathic single organ disorders (e.g., autoimmune pancreatitis, Mikulicz syndrome, and retroperitoneal fibrosis) under one umbrella. It usually presents clinically as tumor-like swelling of the involved organs that can be misdiagnosed as neoplasia. In this case series, we present four cases that were considered as neoplasia but turned out to be IgG4-related disease, we demonstrate the protean manifestations of this condition and variable organs involvement, and we share our experience in using rituximab as the steroid sparing immunosuppressant agent to control this disease.

## 1. Introduction

IgG4-related disease is an emerging multisystemic fibroinflammatory condition that can affect essentially any organ [1]. As its name implies, it is characterized by the presence of abundant IgG4(+) plasma cells in affected tissues, as well as the presence of elevated serum IgG4 concentrations in many patients [2]. It usually manifests as growing soft tissue masses that can closely resemble malignant tumors or lymphomas. Corticosteroids are the mainstay of treatment, but other immunosuppressant agents are also used when the disease is difficult to control. We hereby report four cases with IgG4-related disease which were thought as tumors at the beginning and then diagnosed to have IgG4-related disease.

## 2. Case 1

The first patient is a 34-year-old Bahraini gentleman who was not known to have any medical illness. He had been well until February 2011 when he developed progressive back pain that radiated to his chest wall and upper abdomen associated with significant weight loss. His system review was unremarkable. His past medical history was negative for previous surgeries or medications intake. Socially, he is married and has one daughter. He works as a machine operator in aluminum plant. He smokes one pack of cigarettes daily since age 14. He denied alcohol drinking and illicit drug use. Family history was negative for malignancies and autoimmune diseases.

His laboratory workup including baseline autoimmune workup came back as negative.

**Figure 1:**
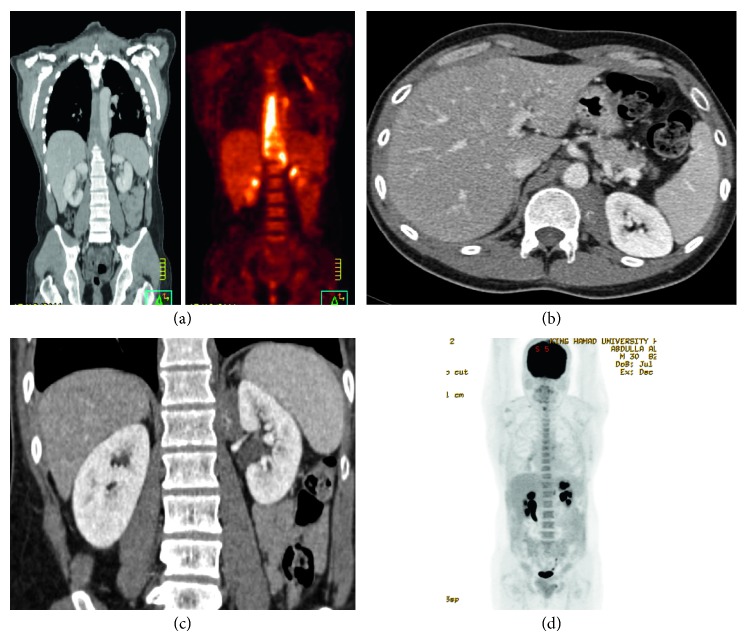
(a) PET CT of 2011 revealed a soft tissue paravertebral mass extending from the T7 till L1. (b, c) Contrast-enhanced computed tomography (CT) scan showing para-aortic soft tissue thickening as well as retrocrural thickening. (d) Bilateral mild hydronephrosis.

Radiographic workup revealed a soft tissue paravertebral mass extending from the T7 till L1 (Figure 1). In April 2011, he underwent left thoracotomy with subtotal resection of the mass. Histopathology showed inflammatory myofibroblastic tumor with reactive lymph nodes. Postoperative PET-CT showed significant residual disease and two hypermetabolic lesions at left pleura and retrocrural tissue.

**Figure 2:**
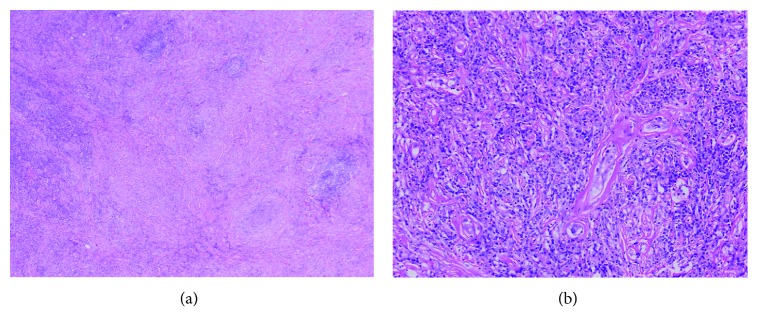
Histological features of retroperitoneal mass in case 1. (a) Low-power magnification shows extensive sclerosis with mixed inflammatory infiltrate and occasional lymphoid aggregates. (b) High-power magnification shows obliterative phlebitis and numerous lymphocytes and plasma cells in the background.

Since the patient did not improve, he was sent abroad for further evaluation. The pathology slides were reviewed again abroad. Due to presence of sclerosing fibrosis (Figure 2(a)) and obliterative phlebitis (Figure 2(b)), IgG4 immunostaining was performed, and it showed moderate numbers of IgG4 plasma cells with a IgG4/IgG plasma cell ratio of >40.

Based on the biopsy findings, he was diagnosed to have idiopathic retroperitoneal fibrosis and IgG4-related disease. He was started on prednisolone and oral cyclophosphamide for 3 months and then maintained on mycophenolate mofetil. In 2015 and 2016, repeated imaging showed disease progression and development of mild bilateral hydronephrosis (Figures 1–1). Therefore, rituximab was given which resulted in significant improvement. His IgG4-level after treatment is 0.604 mg/dl.

## 3. Case 2

A 65-year-old Bahraini female who is a known case of diabetes mellitus, hypertension, and hypothyroid on medical management was doing fine till May/June 2016 when she developed multiple complaints of feeling numbness in the mouth, disrupted sweating over the left side of the face, difficulty in swallowing and clearing mouth secretions, severe intermittent left-sided headaches and facial pain, and multiple episodes of fainting.

Upon close observations of the fainting episodes while being hospitalized, she was found to have sudden loss of consciousness associated with severe sinus bradycardia, sinus pauses, nodal rhythm or complete heart block on some occasions, and hypotension. These episodes were responding to atropine and intravenous fluids. However, later, it got worse, and a pacemaker was inserted.

Upon examination, she was found to have features of Horner's syndrome on the left side of the face, deviation of the tongue to the left side representing left 12^th^ cranial nerve palsy, and a mass observed on the left side of the hard palate. She also had a lobulated, nonmobile mass with smooth margins felt along the left angle of the jaw most likely originating from the left parotid gland.

Her laboratory workup and baseline autoimmune workup including anti-nuclear antibodies (ANAs), extractable nuclear antigens (ENA profile), cytoplasmic anti-neutrophil cytoplasmic antibodies (c-ANCA), and perinuclear anti-neutrophil cytoplasmic antibodies (*p*-ANCA) came back as negative. Her inflammatory markers such as erythrocyte sedimentation rate (ESR) and C-reactive protein (CRP) were low except at times of infection.

**Figure 3:**
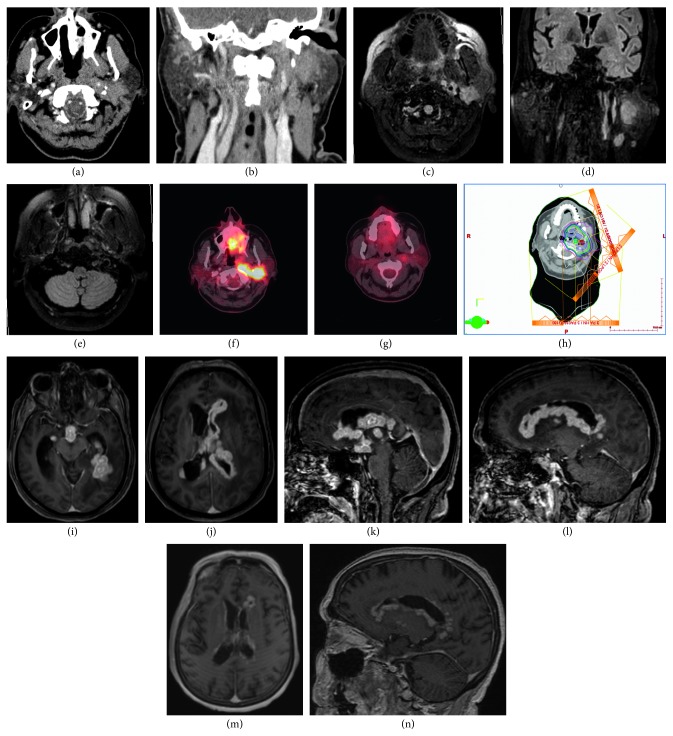
(a, b) Contrast-enhanced CT scan neck showing lobulated enhancing lesion in the deep lobe of the left parotid gland about 2.3 × 2.0 cm. Similar enhancing soft tissue thickening is seen involving adjacent left carotid space reaching up to the base of the skull. (c, d) MRI neck and brain with FLAIR images showing the same lesion in the left parotid gland and carotid space. (e) MRI brain showing involvement of the left 12th cranial nerve at the skull base. (f, g) PET CT presteroid and rituximab (July 2016) and steroid and rituximab (November 2016) showing significant resolution. (h) Proton radiotherapy. (i, j) MRI brain done in August 2017 showing extensive diffuse irregular enhancing subependymal lesions (axial views). (k, l) MRI brain done in August 2017 showing extensive diffuse irregular enhancing subependymal lesions (sagittal views). (m, n) MRI brain done in October 2017 showing significant regression of all lesions after treatment with steroid and rituximab.

The computed tomography scan (CT) and magnetic resonance imaging (MRI) head and neck were done, and they are shown in Figures 3–3:

1. A multilobulated mass within the left parotid gland extending into the deep lobe
2. A similar lesion in the left carotid sheath extending to the base of the skull with anterior extension into the parapharyngeal and pharyngeal mucosal space and the soft palate
3. Cervical lymphadenopathy
4. Involvement of the left 12^th^ cranial nerve at the base of the skull

The positron emission tomography-computed tomography scan (PET-CT) was also performed, and it showed a hypermetabolic soft tissue lesion with a size of 4 cm by 2.3 cm.

Therefore, multiple biopsies (transoral and transcutaneous from multiple sites) were taken from the mass. Histopathology showed sclerosing infiltrative inflammatory pseudotumor, with significant positive staining for IgG4 plasma cells.

Based on the characteristic biopsy findings, she was diagnosed to have IgG4-related disease.

After receiving high-dose steroids followed by rituximab therapy, she had significant improvement and repeated PET-CT after 4 weeks showed significant improvement (Figures 3 and 3). However, unfortunately she lost follow-up and rituximab was stopped by another center.

In February 2017, her symptoms recurred but worse. She became aphasic and was unable to swallow for which she required feeding gastrostomy tube. She went abroad for a second opinion. Her new imaging showed recurrence of the mass. Her pathological slides were reviewed again and diagnosed to have non-Hodgkin's lymphoma like lesion. She was treated with proton therapy form March till May 2017 without significant improvement (Figure 3).

In August 2017, she presented to our hospital with complaints of dizziness and generalized weakness, and then her level of consciousness deteriorated to the level that she required intubation to maintain her airway. Her initial CT brain with contrast was reported as multiple metastatic brain deposits in leptomeninges, suprasellar, and fourth ventricle. So, she was admitted with impression of multiple brain metastasis for evaluation under care of the oncology team.

MRI brain was performed on 29/8/2017 (Figures 3–3). It showed extensive diffuse irregular-lobulated subependymal lesions with restricted diffusion and significant enhancement. Considering the previous medical history of the patient, rheumatology and neurology teams were consulted.

After extensive investigations and taking the whole clinical picture and initial biopsy results into consideration, she was diagnosed as a case of relapse of IgG4-related disease with CNS involvement. Therefore, she was started on high-dose pulse steroid (methylprednisolone) followed by high-dose oral prednisolone and rituximab therapy.

Follow-up MRI brain was done on 5/10/2017 (Figures 3 and 3). It showed good response to treatment with significant regression in previously seen extensive subependymal enhancing lesions with regression in white matter edema. Stable slightly prominent pachymeninges were found. Her IgG4 level was 14.2 mg/dl.

Unfortunately, due to prolonged hospital stay, she suffered from repeated severe, difficult to eradicate health care-associated infections resulting in her death after around 3 months of the admission.

## 4. Case 3

Case 3 is about a 32 year-old Bahraini female. In 1999 (15 years old), she started to have gradual protrusion of both eyes and persistent upper respiratory tract symptoms. Due to the cosmetic effect of the protruding eyes, she was taken to an ophthalmologist by her parents who attributed her symptoms to chronic sinusitis and referred her to an ENT specialist. CT sinuses showed polypoidal masses in all the sinuses. Biopsy showed inflammatory nasal polyps. She was treated with systemic steroids which improved her symptoms significantly and reduced her proptosis. However, once the steroids were tapered, she would flare up again. She also underwent functional endoscopic sinus surgery (FESS) several times to control her condition.

**Figure 4:**
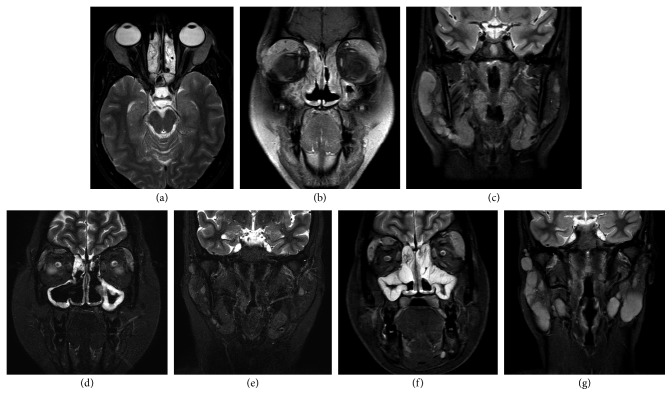
(a, b) MRI head and neck done in May 2013 showing diffuse lacrimal gland enlargement and features of sinusitis. (c) MRI head and neck done in May 2013 showing bilateral parotid and submandibular gland enlargement. (d, e) Follow-up MRI in August 2014 showing significant regression in pan sinusitis, regression in the lacrimal gland, and the salivary gland enlargement after treatment with steroid and azathioprine. (f, g) Follow-up MRI in February 2017 showing disease progression despite steroid and azathioprine.

In 2006 (22 years old), she developed bronchial asthma which was also difficult to control. In 2010 (26 years old), she started to complain of sicca symptoms along with bilateral parotid gland swelling which was investigated by MRI and biopsy. MRI neck and orbits showed the following: bilateral lacrimal glands swelling and enhancement, bilateral parotid and submandibular glands enlargement, multiple intraparotid lymphadenopathy, cervical lymphadenopathy and features of sinusitis (Figures 4–4). Differential diagnosis was kept as possible (Sjögren's syndrome, lymphoma, and sarcoidosis). Parotid gland fine-needle aspiration (FNA) was taken, and it showed reactive lymphoid hyperplasia. No granuloma was found. All serology workup including anti-nuclear antibodies (ANAs), extractable nuclear antigens (ENA profile), cytoplasmic anti-neutrophil cytoplasmic antibodies (c-ANCA), perinuclear anti-neutrophil cytoplasmic antibodies (*p*-ANCA), rheumatoid factor (RF), anti-cyclic citrullinated peptide (anti-CCP), and angiotensin converting enzyme (ACE) level came back as negative. Flow cytometry of fine-needle aspiration (FNA) did not show evidence of lymphoma.

In 2014, the patient decided to go abroad for a second opinion. She underwent parotid gland biopsy and it showed chronic sialadenitis. She was diagnosed to have Mikulicz syndrome and started on steroid and azathioprine. Repeated MRI showed significant response to therapy (Figures 4 and 4).

**Figure 5:**
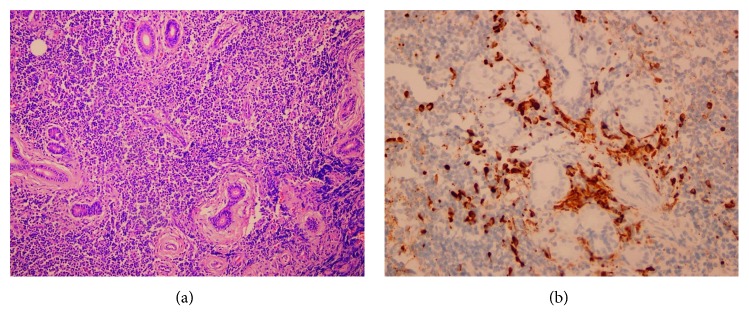
Histological examination of the submandibular gland in case 3. (a) There is dense lymphoplasmacytic infiltrate with acini atrophy and mild periductal fibrosis. No phlebitis seen. (b) IgG4 immunostain occasional clusters of positive plasma cells 10–20 per high-power field.

After 2 years of lost follow-up, she was assessed again when she was admitted for child delivery. Her parotid gland biopsy was reviewed again. It showed patchy dense lymphoplasmacytic infiltrate (Figure 5(a)) with occasional clusters of plasma cells. These plasma cells were mostly positive for IgG4 immunostain (Figure 5(b)) with 10–20 cells per high-power field. No phlebitis was seen. Features were compatible with IgG4-related disease. Serum IgG4 was checked, and it was elevated (3.4 g/L (340 mg/dl)). Therefore, she was diagnosed to have IgG4-related disease. MRI head and neck was repeated on 8/2/2017 (Figures 4 and 4) and showed increase in enlargement of bilateral lacrimal glands, submandibular glands, parotid glands with intraparotid nodes, and cervical lymph nodes by size and numbers. There was also increase in mucosal thickening involving all paranasal sinuses.

Since she is having suboptimal response to azathioprine, rituximab was decided but elected to be postponed by the patient due to fears related to breastfeeding.

## 5. Case 4

A 46-year-old Bahraini female diagnosed as premature ovarian failure at the age of 29 years treated with hormonal replacement therapy presented with a history of epigastric abdominal pain and vomiting at the age of 37 years. Biochemical and radiological assessment showed features of acute pancreatitis in terms of elevated pancreatic enzyme level, and CT abdomen finding showed edematous pancreas with normal ductal system. It was attributed to hormonal replacement therapy after thorough investigation. Although the patient had stopped the implicated medications, she still had recurrent attacks of acute pancreatitis.

Since there was no obvious cause found for her recurrent episodes of pancreatitis, autoimmune pancreatitis was suspected.

**Figure 6:**
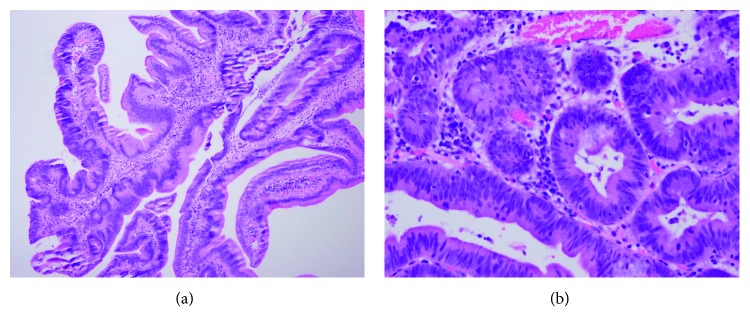
Histological examination of ampullary mass in case 4. (a) Histological examination revealed villotubular structures lined by dysplastic epithelium consistent with adenoma. (b) High-power examination revealed numerous clusters of plasma cells in the intervening lamina propria.

Then, she underwent endoscopic ultrasound in 2015 which revealed mass swelling at the duodenal ampulla, and biopsy was taken. The biopsy showed ampullary adenoma with high-grade dysplasia (Figures 6(a) and 6(b)).

Then, the patient decided to go abroad for further assessment where she underwent Whipple's procedure and histopathology confirmed the presence of ampullary adenoma with high-grade dysplasia.

Unfortunately, she continued to have recurrent episodes of pancreatitis despite the removal of the ampullary adenoma.

In 2016, while she was admitted under care of a surgical team for another episode of pancreatitis, she was reviewed by the rheumatology team to rule out autoimmune condition. Therefore, IgG4 level was tested (1.49 g/L (149 mg/dl)). The biopsy was reassessed and found to have increased IgG4-positive plasma cells around 30–40 per high-power field with the background of adenoma with high-grade dysplasia. Accordingly, she was diagnosed to have both IgG4-related disease and ampullary adenoma.

She was started on oral prednisolone 0.5 mg/kg and rituximab therapy with significant improvement over 1 year of follow-up as the pancreatitis attacks have reduced from around once in every month to around once in every 3 to 4 months after 3 months of rituximab therapy, and currently she remained attack free for around one year.

## 6. Summary of the Cases

The summary of the four cases are presented in Table 1.

## 7. Discussion

Since its first description as an entity by Kamisawa et al. in 2003 [3], IgG4-related disease is increasingly being recognized worldwide. After thorough literature search, this is the first report of IgG4-related disease from Bahrain. Although it usually affects males in their fifth and sixth decades of life [4–10], our patients were mostly females and younger. In fact, the third case had the manifestation at 15 years which is very unusual. The main organs involved are the lymph nodes, salivary glands, lacrimal glands, and pancreas [4–10], which is very similar to our reported cases except the second case which had IgG4-related pachymeningitis which is an extremely rare finding in our literature review. In contrast to most of the cohort studies, our patients had only one major organ involved rather than two.

The diagnosis of this rare condition can be challenging. In 2011, diagnostic criteria were established by a group of Japanese investigators. Having organ dysfunction plus IgG4 plasma level of >135 mg/dl and an IgG4/IgG plasma cell ratio of >40% with >10 IgG4-positive plasma cells per high-power field is diagnostic of IgG4-related disease [11, 12]. However, not all the patients will have elevated serum IgG4. It is elevated in about two-thirds of the patients while the remaining third will have normal level even before treatment and despite the characteristic histopathological findings [9, 13]. The key histopathological findings are dense lymphoplasmacytic infiltrate, fibrosis, arranged at least focally in a storiform pattern, and obliterative phlebitis [1]. Although all our patients had the typical clinical presentations and organ involvement, only cases 3 and 4 had elevated serum IgG4 levels. It was normal and even suppressed in cases 1 and 2 most probably because it was measured only after treatment (rituximab) was initiated. Many reports have shown increased risk of developing malignancies especially pancreatic and non-Hodgkin's lymphoma while others have not [14–17] and is still a matter of debate. Three of our patients were mistaken to have malignancies during the course of their illness.

The first case demonstrates the close resemblance between IgG4-related disease and inflammatory myofibroblastic tumor in terms of clinical presentation and pathological findings [18]. In fact, our patient was diagnosed to have an inflammatory myofibroblastic tumor initially at our hospital, but then the diagnosis was reviewed and changed to IgG4-related disease based on the typical clinical presentation and typical histopathology findings which demonstrate the 3 main features (storiform fibrosis, obliterative phlebitis, and high IgG4/IgG plasma cell ratio of more than 40). Unfortunately, the IgG4 plasma cells staining was done abroad, and we could receive only the report without the slides.

Case 2 was very disputative as it demonstrates the clinical heterogeneity of IgG4-related disease and the difficulty in differentiating it from other infiltrative or inflammatory conditions. The fact that the patient sought at least two medical opinions with different diagnosis and management plans in and outside Bahrain before reaching our hospital and the fact that she had central nervous system (CNS) disease which is a very rare manifestation of IgG4-related disease as mentioned earlier made the diagnostic process very challenging. However, after reviewing all her medical histories and after thorough investigations and long discussion at our multidisciplinary team meeting, we have decided to relate the findings in this patient to IgG4-related disease rather than other infiltrative, inflammatory, or neoplastic conditions such as lymphoma or vasculitis which were contemplated as differential diagnosis but were ruled out.

Although she presented to us with CNS manifestations, her initial presentation was left parotid gland mass extending to infiltrate the left carotid sheath. A biopsy was taken, and it showed characteristic features of IgG4-related disease. Unfortunately, we could not get the histology slides. Based on the clinical presentation and biopsy findings, she was started on rituximab therapy and she responded very well initially. However, after stopping rituximab by another center, she developed new CNS lesions. The lack of sinuses, lungs, and kidneys involvement at presentation makes anti-neutrophil cytoplasmic antibodies (ANCA) associated vasculitis unlikely as a differential diagnosis. In addition, inflammatory markers (erythrocyte sedimentation rate (ESR) and C-reactive protein (CRP)) were not elevated and both cytoplasmic anti-neutrophil cytoplasmic antibodies (c-ANCA) and perinuclear anti-neutrophil cytoplasmic antibodies (*p*-ANCA) were negative. Furthermore, the biopsy specimen lacks features of ANCA associated vasculitis such as necrotizing granuloma formation. Considering the clinical presentation, lymphoma was on the top of our differential diagnosis, but extensive workup of the lesion ruled out lymphomatous histology with surface markers were all negative for lymphoma.

In case 3, the diagnosis of IgG4-related lacrimal and salivary gland disease was based on the typical organ involvement evidenced by the clinical presentation and the radiologic imaging, high IgG4 level 340 mg/dl, and biopsy findings. Unfortunately, the histopathological specimen was taken from our patient after she had received multiple high-dose steroid courses to which she responded clinically and resulted in significant reduction in glands swelling. Thus, the reported low IgG4-positive plasma cells (10 to 20 per high-power field) which are less than the typical number required to diagnose IgG4-related lacrimal and salivary gland disease (>100 IgG4-positive plasma cells per high-power field [1]) might be related to the partial or complete response to steroid therapy, and we recognize this as a limitation in the diagnostic process of this case.

In case 4, the diagnosis of type 1 autoimmune pancreatitis or IgG4-related pancreatitis was based on the following observations.

The patient presented with recurrent attacks of acute pancreatitis manifested by epigastric pain, vomiting, and elevated levels of pancreatic enzymes and positive CT findings. The first CT abdomen was done in 2007 when she had her first attack. It showed diffuse edematous enlarged pancreas with normal biliary system and pancreatic duct which is consistent with acute pancreatitis. Unfortunately, we have only the report as the images were lost upon changing the whole computer system of the hospital. According to the International Consensus Diagnostic Criteria for Autoimmune Pancreatitis published in 2011 [19], ampullary biopsy showing abundant IgG4-positive plasma cells (>10 cells per high-power field) can be used if pancreatic tissue is not available for diagnosing autoimmune pancreatitis if other criteria are met, which is the case in our patient. Furthermore, her remarkable response to steroid treatment supports the diagnosis of autoimmune pancreatitis. Therefore, although this patient is diagnosed to have ampullary adenoma with high-grade dysplasia, she still fits the whole three criteria required for IgG4-related disease (a typical organ involvement, IgG4 level of 149 mg/dl (>135) and positive histopathology and an excellent response to steroid therapy). Although this might be a matter of debate, we strongly believe based on the above findings that this patient is having both ampullary adenoma with high-grade dysplasia and IgG4-related autoimmune pancreatitis.

The four cases were treated with steroid which is consistent with the International Consensus Guidance Statement on the Management and Treatment of IgG4-Related Disease published in 2015 [20]. All patients have responded very well to steroids. Since CNS involvement holds grave prognosis, case 2 received methylprednisolone pulse therapy 1 g intravenously for 3 days followed by prednisolone 1 mg per kg. In addition, rituximab therapy was given as additional immunosuppressive therapy and steroid sparing agent which resulted in almost full clearance of the brain lesions. The other cases also had excellent response to steroid therapy which supports the diagnosis of IgG4-related disease. Three of the four cases were treated with rituximab therapy using one gram on day 0 and another one gram on day 15 protocol. Although formal assessment using the IgG4 RD responder index [21] was not done, all the treated cases showed clinical improvement and decrease in relapse rate with rituximab therapy adding to the existing evidence that rituximab is a promising therapy as the steroid sparing immunosuppressive agent in IgG4-related disease [22–27].

## 8. Conclusion

IgG4-related disease is a complex systemic disease of unknown etiology. It usually presents as growing soft tissue masses that can affect virtually any organ in the body. Differentiating IgG4-related disease from malignancies can be difficult on many occasions. Steroids and rituximab therapy seem to be effective therapy for controlling the disease.

## Figures and Tables

**Table 1:** 

Case	Clinical presentation	Initial diagnosis	Treatment	Serum IgG4 level
1	Paravertebral mass	Inflammatory myofibroblastic tumor Idiopathic retroperitoneal fibrosis	Oral cyclophosphamide Mycophenolate mofetil Steroid and rituximab	0.604 mg/dl (after treatment) Pretreatment level is not available
2	Parotid mass Pachymeninges	Inflammatory pseudotumor Non-Hodgkin's lymphoma	Steroid and rituximab Proton therapy	14.2 mg/dl after steroid treatment Pretreatment level is not available
3	Proptosis Lacrimal and salivary gland swelling	Mikulicz syndrome	Steroid and azathioprine	340 mg/dl Pretreatment level
4	Recurrent pancreatitis	Ampullary adenoma	Whipple's procedure Steroid and rituximab	149 mg/dl Pretreatment level

## Additional Points

*Learning Points.* Due to the nature of presentation of IgG4-related disease as growing soft tissue masses, differentiating it from malignancies is crucial and can be challenging in many occasions. B-cell depletion therapy is a promising modality of treatment. However, large randomized trials are needed.

## References

[1] Deshpande V., Zen Y., Chan J. K. C. (2012). Consensus statement on the pathology of IgG4-related disease. *Modern Pathology*.

[2] Della-Torre E., Lanzillotta M., Doglioni C. (2015). Immunology of IgG4-related disease. *Clinical and Experimental Immunology*.

[3] Kamisawa T., Funata N., Hayashi Y. (2003). A new clinicopathological entity of IgG4-related autoimmune disease. *Journal of Gastroenterology*.

[4] Lin W., Lu S., Chen H. (2015). Clinical characteristics of immunoglobulin G4-related disease: a prospective study of 118 Chinese patients. *Rheumatology*.

[5] Campochiaro C., Ramirez G. A., Bozzolo E. P. (2015). IgG4-related disease in Italy: clinical features and outcomes of a large cohort of patients. *Scandinavian Journal of Rheumatology*.

[6] Yamada K., Yamamoto M., Saeki T. (2017). New clues to the nature of immunoglobulin G4-related disease: a retrospective Japanese multicenter study of baseline clinical features of 334 cases. *Arthritis Research and Therapy*.

[7] Fernandez-Codina A., Martinez-Valle F., Pinilla B. (2015). IgG4-related disease: results from a multicenter Spanish registry. *Medicine*.

[8] Inoue D., Yoshida K., Yoneda N. (2015). IgG4-related disease: dataset of 235 consecutive patients. *Medicine*.

[9] Wallace Z. S., Deshpande V., Mattoo H. (2015). IgG4-related disease: clinical and laboratory features in one hundred twenty-five patients. *Arthritis and Rheumatology*.

[10] Sekiguchi H., Horie R., Kanai M., Suzuki R., Yi E. S., Ryu J. H. (2016). IgG4-related disease: retrospective analysis of one hundred sixty-six patients. *Arthritis and Rheumatology*.

[11] Umehara H., Okazaki K., Masaki Y. (2012). Comprehensive diagnostic criteria for IgG4-related disease (IgG4-RD), 2011. *Modern Rheumatology*.

[12] Umehara H., Okazaki K., Nakamura T. (2017). Current approach to the diagnosis of IgG4-related disease-combination of comprehensive diagnostic and organ-specific criteria. *Modern Rheumatology*.

[13] Carruthers M. N., Khosroshahi A., Augustin T., Deshpande V., Stone J. H. (2015). The diagnostic utility of serum IgG4 concentrations in IgG4-related disease. *Annals of the Rheumatic Diseases*.

[14] Hirano K., Tada M., Sasahira N. (2014). Incidence of malignancies in patients with IgG4-related disease. *Internal Medicine*.

[15] Asano J., Watanabe T., Oguchi T. (2015). Association between immunoglobulin G4-related disease and malignancy within 12 years after diagnosis: an analysis after longterm followup. *Journal of Rheumatology*.

[16] Yamamoto M., Takahashi H., Tabeya T. (2011). Risk of malignancies in IgG4-related disease. *Modern Rheumatology*.

[17] Takahashi N., Ghazale A. H., Smyrk T. C., Mandrekar J. N., Chari S. T. (2009). Possible association between IgG4-associated systemic disease with or without autoimmune pancreatitis and non-Hodgkin lymphoma. *Pancreas*.

[18] Yamamoto H., Yamaguchi H., Aishima S. (2009). Inflammatory myofibroblastic tumor versus IgG4-related sclerosing disease and inflammatory pseudotumor: a comparative clinicopathologic study. *American Journal of Surgical Pathology*.

[19] Shimosegawa T., Chari S. T., Frulloni L. (2011). International consensus diagnostic criteria for autoimmune pancreatitis: guidelines of the international association of pancreatology. *Pancreas*.

[20] Khosroshahi A., Wallace Z. S., Crowe J. L. (2015). International consensus guidance statement on the management and treatment of IgG4-related disease. *Arthritis and Rheumatology*.

[21] Carruthers M. N., Stone J. H., Deshpande V., Khosroshahi A. (2012). Development of an IgG4-RD responder index. *International Journal of Rheumatology*.

[22] Khosroshahi A., Bloch D. B., Deshpande V., Stone J. H. (2010). Rituximab therapy leads to rapid decline of serum IgG4 levels and prompt clinical improvement in IgG4-related systemic disease. *Arthritis and Rheumatology*.

[23] Khosroshahi A., Stone J. H. (2011). Treatment approaches to IgG4-related systemic disease. *Current Opinion in Rheumatology*.

[24] Khosroshahi A., Carruthers M. N., Deshpande V., Unizony S., Bloch D. B., Stone J. H. (2012). Rituximab for the treatment of IgG4-related disease: lessons from 10 consecutive patients. *Medicine*.

[25] Sedyshev S. K., Vasil’ev V. I., Kovrigina A. M. (2013). IgG4-related disease: patient group characterization and rituximab therapy. *Terapevticheskii arkhiv*.

[26] Ebbo M., Grados A., Samson M. (2017). Long-term efficacy and safety of rituximab in IgG4-related disease: data from a French nationwide study of thirty-three patients. *PLoS One*.

[27] Carruthers M. N., Topazian M. D., Khosroshahi A. (2015). Rituximab for IgG4-related disease: a prospective, open-label trial. *Annals of Rheumatic Diseases*.

